# Chimpanzee-Guided Discovery of a Non-Native Bioactive Plant

**DOI:** 10.3390/ani16132031

**Published:** 2026-07-02

**Authors:** Sabrina Krief, Hugo Magaldi, Myriam Kourdourli, Marc Jeanson, Raymond Katumba, Harold Rugonge, John Justice Tibesigwa, Marc Litaudon, Florent Olivon

**Affiliations:** 1 Eco-Anthropologie et Ethnobiologie UMR 7206 CNRS/MNHN/Université Paris 7, Hommes et Environnement, Museum National d’Histoire Naturelle, Musée de l’Homme, 75016 Paris, France; hugo.magaldi@mnhn.fr; 2 Sebitoli Chimpanzee Project, Great Ape Conservation Project (GACP), Sebitoli Research Station, Kibale National Park, Fort Portal, Uganda; rmuhoja94@gmail.com (R.K.); hrugonge@gmail.com (H.R.); 3 Direction Générale Déléguée Aux Collections, Direction des Collections Naturalistes, Anthropologie Culturelle, Ethnobotanique (DGDC—DCN), Muséum National d’Histoire Naturelle, CP 50, 75005 Paris, France; myriam.kourdourli@mnhn.fr; 4 Institut de Systématique, Évolution, Biodiversité (ISYEB), UMR 7205 CNRS/MNHN/UPMC EPHE, Muséum National d’Histoire Naturelle, CP 50, 75005 Paris, France; marc.jeanson@mnhn.fr; 5 Uganda Wildlife Authority, Plot 7 Kira Road, Kampala P.O. Box 3530, Uganda; jjtibesigwa@gmail.com; 6 Institut de Chimie des Substances Naturelles, CNRS, UPR 2301, Université Paris-Saclay, CNRS, 91198 Gif-surYvette, France; marc.litaudon@cnrs.fr (M.L.); florent.olivon@cnrs.fr (F.O.)

**Keywords:** animal self-medication, chimpanzee, pharmacological properties of plants, withanolides, tropical forests

## Abstract

Studies on chimpanzees suggest that some plants are consumed for their biological properties. Low frequency of consumption, small ingested quantities, complex food processing for a low nutritional value, and local ecological knowledge from human populations can serve as useful indicators for detecting self-medicative behavior. Since 2008, wild chimpanzees have been monitored in Sebitoli, Kibale National Park, Uganda. This study focuses on a plant locally called “Angel’s trumpet,” selectively consumed by chimpanzees but previously botanically unidentified. Across 890 observation days, 25 consumption events were recorded involving 18 chimpanzees, each ingesting about 10 g of pith. The plant was botanically identified as *Acnistus arborescens*, a species native to Central and South America and not previously reported in Africa. The plant is known to contain withanolides particularly studied in mice and humans for their anxiolytic effects. Chemical analysis of leaves and pith conducted during this survey revealed diverse withanolides and cinnamides, with some compounds unique to the pith. This finding highlights a chimpanzee-guided discovery of a non-native plant with known bioactive properties. This study opens avenues for further research on its distribution, potential invasiveness, and potential uses for its biological effects by both humans and wildlife in Africa.

## Simple Summary

Studies on chimpanzees suggest that they may sometimes eat certain plants, because of their health benefits. Rare use, small amounts eaten, and unusual ways of processing low-nutritional plants can help to identify self-medicative behaviors. Since 2008, wild chimpanzees have been studied in Kibale National Park in Uganda. These chimpanzees occasionally eat a plant known locally as “Angel’s trumpet,” though its exact identity was unknown. This study identified the plant as a species originally from the Americas that had not been reported in Africa before. It also confirms the presence of compounds known for anxiolytic properties tested in mice and humans. The chimpanzees eat only a small amount of the inner part of the plant’s stem, suggesting they are not using it as food but possibly for a specific effect. This behavior indicates that animals may help us discover plants with useful properties. Further research may focus on its presence in Africa, its effects on humans and animals, and its potential environmental impact.

## 1. Introduction

Studies on great apes and more specifically on chimpanzees (*Pan troglodytes*) suggest that the use or the ingestion of certain plants is motivated by their benefits on health rather than being a by-product of feeding behavior [1,2,3,4,5,6,7,8,9,10,11]. During field observations, evidence supporting self-medication in chimpanzees includes the occasional consumption of plant species that do not constitute a regular component of their diet, the ingestion of plants with little or no apparent nutritional value, and the selective use of these plants during illness (cough, diarrhea, injury, etc.) or specific seasons such as periods with high intestinal parasite load [1,12,13]. These consumptions differ from feeding behaviors by their low frequency, the small quantities ingested, and the particular food-processing techniques involved. For example, rough leaves are swallowed whole without mastication, while bark is stripped from trees through prolonged effort despite its apparently low nutritional value [14,15,16]. For a behavior to be reliably classified as self-medicative, the plant material consumed should have physical properties (trichomes of rough leaves) or chemical properties, either already documented or revealed through chemical characterization and biological assays demonstrating their therapeutic potential [1,8,17,18]. The most compelling studies demonstrate that the consuming individual exhibited a pathological condition consistent with the pharmacological properties of the ingested item, consumed it in an amount likely to produce a therapeutic effect, and subsequently showed recovery associated with its consumption [1]. Nevertheless, implementing such health monitoring and diagnostic procedures are not always practically feasible, particularly under field conditions [19]. Strict ethical guidelines and sanitary regulations for research on a threatened species such as the chimpanzee prevent any invasive investigation and are constraints faced by researchers. Visual monitoring and urine or fecal analysis cannot always provide objective evidence for internal state motivating ingestion of bioactive natural products (nausea; depression; anxiety; stress; fever; internal pain, such as headache; stomach pain; etc.).

Other indicators may be used to identify self-medication behaviors such as independent convergent uses by people and animals [20,21,22,23]. Local uses and knowledge associated with these plants or natural substances have, in some cases, led to the identification of novel compounds [6,20,21,22,23].

In addition to offering solutions aimed at optimizing the health of people, animals, and ecosystems in a One Health approach, such an investigation may help to better understand the origin of human medicine, as well as the intrinsic capacities and knowledge of nonhuman animals [20,24,25].

In Sebitoli, in the northern sector of Kibale National Park, chimpanzees have been monitored daily by a research team since 2008. The consumption of a plant locally known as “Angel’s trumpet” was first observed and recorded in 2022, fourteen years after the initiation of their monitoring. The research team was surprised by this consumption, as the plant is locally considered as toxic. The ingestion was limited to small amounts of the inner stem of the plant. A multidisciplinary survey was therefore undertaken to identify the plant species and test several hypotheses: (1) the consumption resulted from error or a random behavior, in which case it would not recur or it would occur in young or immigrant individuals who were inexperienced; (2) the consumption fulfilled a nutritional function, and in that case, large amounts may be consumed in some periods; or (3) this plant part has specific biological properties, and in that case, chemical investigation will inform us about the presence of compounds known for their pharmacological properties, the frequency and the amount of the plant item consumed will be low, and associated symptoms could be recorded. To test this latter hypothesis, we compared the annual consumption frequency of this item with that of natural substances known to be used for self-medication in the study area. The present study therefore combines botanical identification, phytochemical analyses, and behavioral and health monitoring to test these hypotheses.

## 2. Material and Methods

### 2.1. Study Site

Kibale National Park is located in Southwestern Uganda between 0°13′–0°41′ N and 30°19′–30°32′ E. The 795-km^2^ park was established in 1993 and is currently under the management of the Uganda Wildlife Authority (UWA). The protected area is mostly covered by moist evergreen or semi-deciduous forest, at various stages of maturity and regeneration from past human disturbance [26]. The Sebitoli area covers 25 km^2^ located at the extreme north of Kibale National Park. The Sebitoli forest was commercially logged in the 1970s, causing damage to about 50% of the trees [27]. Today, 70% of the Sebitoli area is made up of regenerating forests, and only 14% of it consists of old-growth forests [28]. More than 80% of the perimeter of the home range of the Sebitoli community is in contact with the anthropogenic landscape (mostly farmland and tea estates) [28]. Batooro and Bakiga are the main ethnic communities in the area, and Batooro is particularly represented in the north of the park [22].

### 2.2. Botanical Determination and Ethnomedicinal Use

The voucher specimens of the Angel’s trumpet were collected from the Sebitoli area for both phytochemical and botanical surveys at the location 36N0209244 UTM 0072758. The voucher specimen (number P01164692) was determined at the MNHN Herbarium.

### 2.3. Chimpanzee Observation

Approximately 80–100 chimpanzees inhabit a 25 km^2^ area in the northern part of the park called Sebitoli. Since 2008, they have been censused, habituated, individually identified with names and codes, and studied by the Sebitoli Chimpanzee Project [29,30]. The food items consumed by these chimpanzees have been systematically recorded, leading to the establishment of a list of their usual foods.

The fission–fusion social structure of chimpanzees makes it impossible to monitor all community members at once. To address this, two (Sundays, public holidays), three (Fridays and Saturdays), or four (weekdays) teams of two Ugandan field assistants, all originating from the vicinity and being Batooro and Bakiga, track the chimpanzees daily, focusing on subgroups known as “parties”. Each morning, data collection begins as soon as the chimpanzees are located, ideally around 6:30–7:00 am, when they wake up, and their identities are confirmed, and it stops when the chimpanzees are nesting, around 6:30–7:00 pm. Two teams start at 5:00 am to reach chimpanzee nests or look for them in case they were not nested the previous day, while two teams start at 10:30 am and will follow the chimpanzees until nesting time. On average, daily observations of chimpanzees cumulate 20 h for a mean party size of 5 individuals, covering about 89,000 h of observation during the 890 days of the study period (20 × 890 × 5 chimpanzees). Systematic data are then collected, including party composition and focal animal sampling [31]. Ad libitum data are gathered when specific behaviors, such as the consumption of rarely eaten items, are observed, and the ingested amount is estimated. In the case of the pith of the Angel’s trumpet and the inner part of the woody stems, 5 samples of each were measured and weighed to estimate the weight of the fresh part consumed by the chimpanzees. Additionally, chimpanzee tracks and locations where specific behaviors occur are recorded using a GPS 66sr^TM^ Garmin (Olathe, KS, USA). A daily health check is conducted on each individual in the observed party. For 15 min, when the individual is clearly visible, any symptoms of respiratory (sneezing and coughing) or digestive (constipation, diarrhea, etc.) illnesses are monitored. In addition, every part of their body is examined for potential wounds, ophthalmological problems, or locomotor disorders (for more details, see [19]). Considering the average body weight for *Pan troglodytes schweinfurthii* for adult males (42 kg) and adult females (35 kg; [32]), and for subadult, juvenile, and infant individuals, respectively, 30, 20, and 5 kg (SCP, unpublished data obtained after weighing anesthetized snared individuals), we estimated the average weight of a consumer.

### 2.4. Estimation of the Annual Consumption Frequency

To test the low-consumption hypothesis, we estimated the individual annual consumption frequency (i.e., the number of consumptions per individual and per year) of Angel’s trumpet. For that purpose, we recorded the number of observed consumptions of Angel’s trumpet (*x*) and their average duration (*d*) in min. We then formalized an event as a time period of duration, d, during which consumption can occur at 0 or 1 time. Consumption was therefore assessed at the event level, and consecutive minutes within the same event were not treated as independent observations.

The number of observed events (*n*) was deduced from the total observation time (in min) divided by the duration of the event, *d* (in min). We modeled the observed events, *X*_1_, …, *X_n_*, with *X_i_* = 1 if consumption occurred during the event, and *X_i_* = 0 otherwise. We assumed that *X_i_* values were independent and identically distributed (i.i.d.) with a Bernoulli distribution of parameter *p*_0_, where *p*_0_ is the probability of consumption per event. This i.i.d. assumption is a modeling simplification: it presumes similar consumption behavior across individuals such that variation in sex and age among chimpanzees, and thus the composition of observed parties, is not explicitly accounted for in the model. The parameter *p*_0_ was estimated by the proportion *p* of events with consumption: *p* = ∑_i=1,…,*n*_*X_i_*/*n* = *x*/*n*. The uncertainty was quantified using the central limit theorem for *p* with a plug-in variance estimate, *σ*^2^ = *p*(1 − *p*) (Slutsky’s theorem), and the associated standard error (*SE*) = *σ*/sqrt(*n*), yielding a 95% confidence interval [*p* − 1.96*SE*, p + 1.96*SE*].

To estimate the average annual number of Angel’s trumpet consumptions for one chimpanzee, we assumed that events could occur only during daylight (12 h/day in the study area located near the Equator, using the web tool [33]), and we computed the total annual number of events *m* = 365 × 12 × 60/*d*. Therefore, the number of annual consumptions for one chimpanzee was modeled as *N_year_* ~Binomial(*m*,*p*), a binomial distribution of the parameters m and p, and we calculated its mean value, E [*N_year_*] = *m* × *p*.

We compared this number of consumptions with the other unusual consumptions of items that have demonstrated bioactive properties and that have low nutritive value, already recorded in chimpanzees in Kibale National Park: *Trichilia rubescens* leaves [5], *Albizia grandibracteata* bark and wax [8], rough leaves of *Aneilema aecquinoctiale* and *Rubia cordifolia* swallowing [6], and soil consumption [34] over the same year (2024). We calculated the estimated annual number of consumptions for *Trichilia rubescens*, *Albizia grandibracteata*, and rough leaves using the same methodology as for *A. arborescens*. We did not calculate the number of soil consumption events using the same method, as the collection and quantities of soil ingested are very low, last only a few seconds, and do not require any process.

### 2.5. Phytochemistry

#### 2.5.1. Plant Collection

The stems and leaves of the Angel’s trumpet were collected at two different locations where the consumption by Sebitoli chimpanzees was observed. Fresh leaves were collected and cut into small pieces < 2 cm. The stems were opened in two parts, and the very small part inside the stems that was observed to be eaten by chimpanzees was collected and cut into small pieces. The leaves and parts of the stem samples inside were dried in a ventilated room on a mosquito net, without exposure to direct sunlight. LC-MS and molecular network analysis were conducted at the ICSN-CNRS laboratory.

#### 2.5.2. Extraction of Plants and Preparation of Samples

Approximately 5 g of leaves and 5 g of pith were extracted three times for 1 h at room temperature with ethyl acetate (EtOAc) (100 mL each). The plant residues were then re-extracted three times for 1 h at room temperature with methanol (MeOH) (100 mL each). EtOAc and MeOH extracts were concentrated in vacuo at 40 °C. Samples were prepared at 1 mg·mL^−1^ in H_2_O-MeOH-DMSO, 70:20:10, and then diluted to 0.2 mg·mL^−1^ in H_2_O-MeOH, 90:10.

#### 2.5.3. Data-Dependent LC-ESI-HRMS2 Analyses

Liquid chromatography (LC) analyses were performed using a Waters ACQUITY UPLC I-Class system equipped with a Cortecs C18 column (2.1 × 100 mm; 2.7 µm, Waters, Guyancourt, France). The mobile phase consisted of water–acetonitrile (H2O-CH3CN) acidified with 0.1% formic acid (90:10) kept for 2 min, followed by a gradient from 90:10 to 0:100 over 20 min held at 0:100 for 8 min at a flow rate of 600 µL·min^−1^. The injector temperature was set at 10 °C, the column oven was set at 40 °C, and the injection volume was 2 µL.

Electrospray–high-resolution tandem mass spectrometry (ESI-HRMS2) data were collected on a Waters Vion IMS Q-ToF mass spectrometer (Waters Corporation, Wilmslow, UK/Wexford, Ireland) operating in positive-ion mode and controlled by Waters_Connect 4.2.0 software. The source parameters were set as follows: capillary voltage, 1 kV; cone voltage, 20 V; source offset voltage, 80 V; source temperature, 120 °C; desolvation temperature, 500 °C; cone gas flow rate (N2), 100 L·h^−1^; and desolvation gas flow rate (N2), 1000 L·h^−1^. MS1 scans were operated in full-scan mode from *m*/*z* 100 to 1200 (scan time = 0.1 s), followed by MS2 scans of the five most intense ions above an absolute threshold of 10,000 counts per second. The selected precursor ions were fragmented using a ramp collision energy of 20–40 eV to 30–60 eV as a function of the precursor *m*/*z* value and using an *m*/*z*-dependent isolation window of 2–4 amu. Data calibration was performed using an external reference (LockSpray) by constant infusion of leucine-enkephalin solution (200 ng·mL^−1^) at 20 μL·min^−1^.

#### 2.5.4. Pre-Processing of LC-MS Data

Raw data were converted to Lockmass-corrected and centroided .mzML data files using the DATA Convert module, part of the Waters_Connect software. All .mzML files were modified using the precursor *m*/*z* value corrector provided by Breaud et al. [35] and then processed using MZmine 4.4.3 [36,37,38]. Detailed MZmine processing parameters are provided in Supplementary Materials.

Eventually, the .mgf and .csv files needed for subsequent molecular networking and annotation processing were exported using the dedicated modules “Molecular networking files” and “Export for SIRIUS”. The mzML files of the plant extracts have been deposited in the MassIVE data repository (MassIVE ID: MSV000098772).

#### 2.5.5. Compound Annotations

Metabolite annotation was performed in MetGem by searching for GNPS spectral library matches [39,40]. Additionally, molecular formulae, chemical structures, and compound classes were annotated using Sirius 6.0.7 [41,42,43,44,45,46]. Detailed Sirius processing parameters are provided in Supplementary Material.

#### 2.5.6. Analysis of the Molecular Network

The .mgf spectral data and .csv files for molecular networking were imported into MetGem 1.5.2 [47,48]. The m/*z* tolerance window used to find matching peaks was set to 0.005 Da, and cosine scores were calculated for spectra sharing at least 5 matching peaks. The MSMS spectra were filtered, keeping peaks higher than 50 Da and choosing the top 10 peaks within the ±50 Da window throughout the spectrum. Standard molecular networks were created in which edges were filtered to have a cosine score greater than 0.7. Further edges between two nodes were retained in the network only if each of the mutual MSMS spectra appeared in each other’s respective top 10 most similar spectra.

The network output was imported into Cytoscape 3.10.2 for advanced network visualization and image export [49].

## 3. Results

### 3.1. Botanical Determination and Ecology of Plant Species in Sebitoli, Uganda

**Figure 1:**
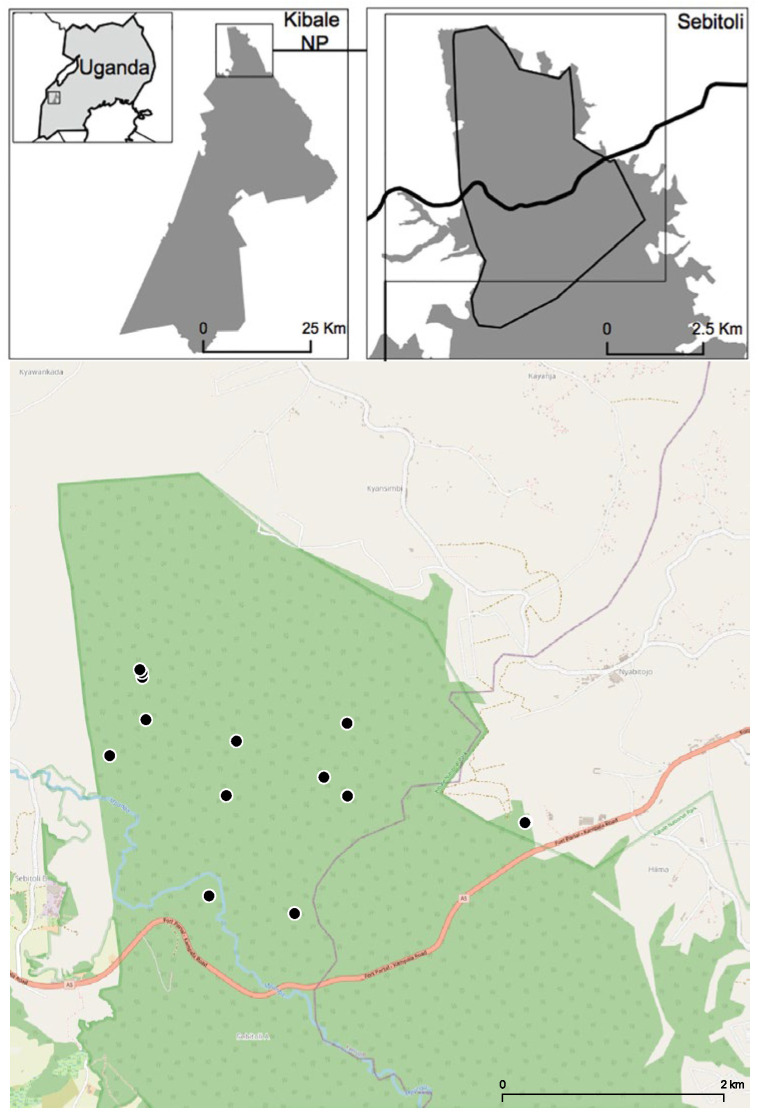
Location of the observed consumptions of *Acnistus arborescens* in the home range of the Sebitoli chimpanzee community. Sebitoli is located in the northern part of the Kibale National Park, Uganda.

Only 10 locations of this plant species are known so far by teams working daily in this area of Sebitoli since 2008 (Figure 1). The plant is a shrub to small tree, bearing bell-shaped flowers with white corolla clustered along the stem. In its native range, this species is characteristic of montane cloud forests (in clearings, forest edges, etc.) at elevations between 300 and 2000 m. The SCP team also planted a small specimen at the research station within the forest to monitor the seasonal flowering and fruiting, but no fruits were produced in two years. After close morphological observation and comparison with physical and digitized herbarium collections, MJ identified the voucher specimen of a blooming individual as a Solanaceae species named *Acnistus arborescens* (L.) Schltdl. Linnaea 7:67. 1832 (syn. *Iochroma arborescens*, J.M.H. Shaw).

A specimen was deposited in the vascular plant collection (P) of the Herbarium at MNHN. This sample is visible in the MNHN vascular plant collection [50], and one duplicate was sent to the Kew Royal Botanical Garden Herbarium (K). The plant name has been checked with The Plant List [51].

### 3.2. Chimpanzee Behavior Related to Angel’s Trumpet

The first observation of *A. arborescens* pith by a Sebitoli chimpanzee occurred on 9 July 2022. The alpha male of the community, Elliott, 45 years old, consumed approximately 8 cm of the inside part of a stem for 12 min. Following this initial observation of the alpha-male consumption, an additional 24 consumptions were recorded in 890 days, between 9 July 2022 and 15 December 2024 (Supplementary Material Table S1).

During 22 of these episodes, the consumer was identified. A total of 18 different individuals (10 males and eight females) consumed stems of *A. arborescens*. An adult female (KE) consumed the pith or inner part of the stem on three different occasions. For four individuals (three females and one male), two consumption episodes were recorded, while all other individuals consumed the plant only once. In three instances, observers arrived when the stems had already been consumed, making it impossible to determine which chimpanzee(s) had eaten what quantity. Of the 25 episodes, nine involved a single chimpanzee consuming the plant while in a party of multiple individuals (mean party size = 9.6 individuals), and four episodes saw multiple individuals consuming the plant, typically involving a female with her infants (females KE, SA, MJ, and KT). The average duration of the process of opening or peeling the stems and consuming them was 6 min and 50 s, with a range of 34 s to 22 min, and most (17 out of 25) occurred in the afternoon. The cumulative duration of stem processing and consumption reached 3 h, 24 min, and 50 s out of 89,000 h of observation.

In 2024, out of 36,500 h of observation, recorded ingestions of other items of low or no nutritional value comprised 20 cases of *A. arborescens* (6 min 9 s), 118 cases of soil consumption, 32 cases involving *Trichilia rubescens* (mean duration = 14 min, 13 leaves ingested), six cases of *Albizia grandibracteata* bark (mean duration = 1 min 24 s) and wax, and seven cases of rough-leaf swallowing (mean duration = 1 min 11 s). We report the estimated yearly consumptions of *Acnistus arborescens*, *Trichilia rubescens*, *Albizia grandibracteata*, and rough leaves (Table 1).

**Scheme 1:**
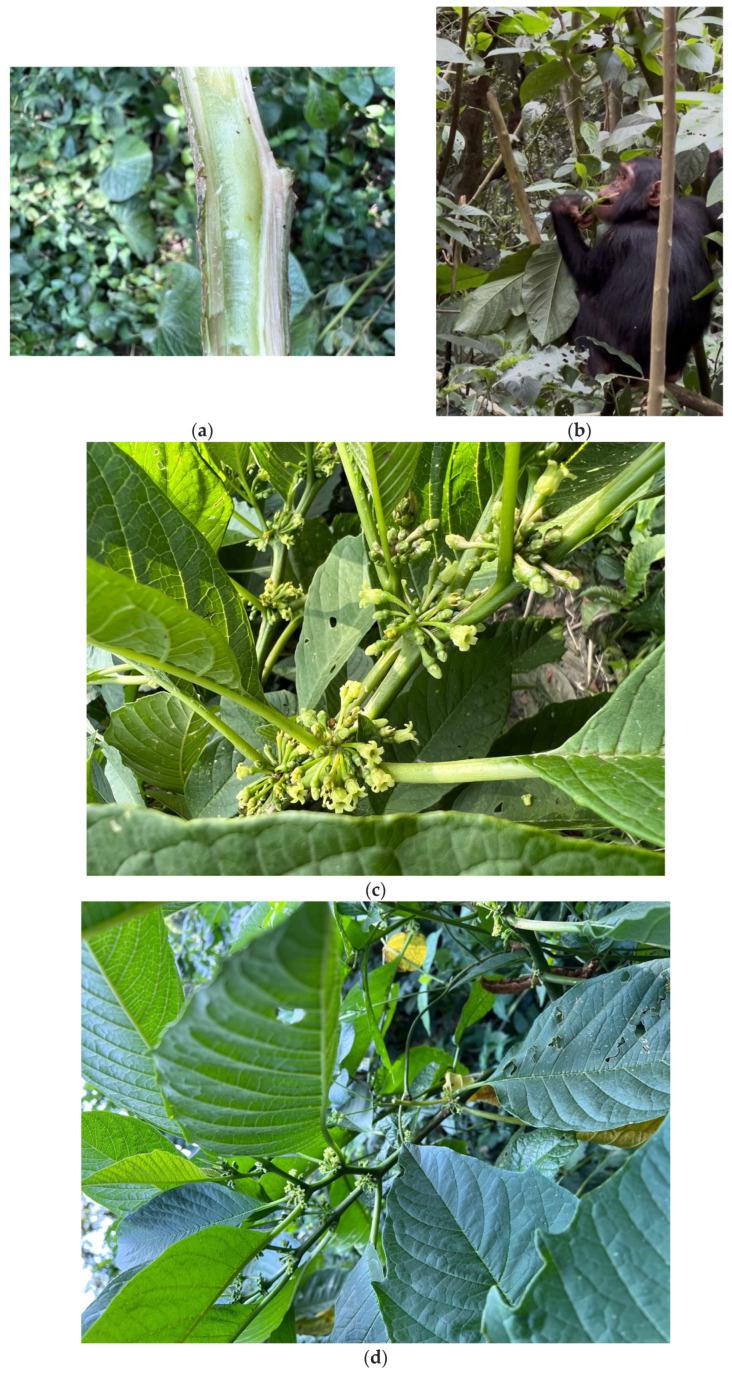
(**a**) Inner part of a stem of *Acnistus arborescens* targeted by chimpanzees (credit: Sabrina Krief/Sebitoli Chimpanzee Project). (**b**) Juvenile chimpanzee KN feeding young pith of *Acnistus arborescens* (credit: Sabrina Krief/Sebitoli Chimpanzee Project). (**c**,**d**) *Acnistus arborescens* flowering at Sebitoli, Kibale National Park, Uganda (credit: Sabrina Krief/Sebitoli Chimpanzee Project).

**Scheme 2:**
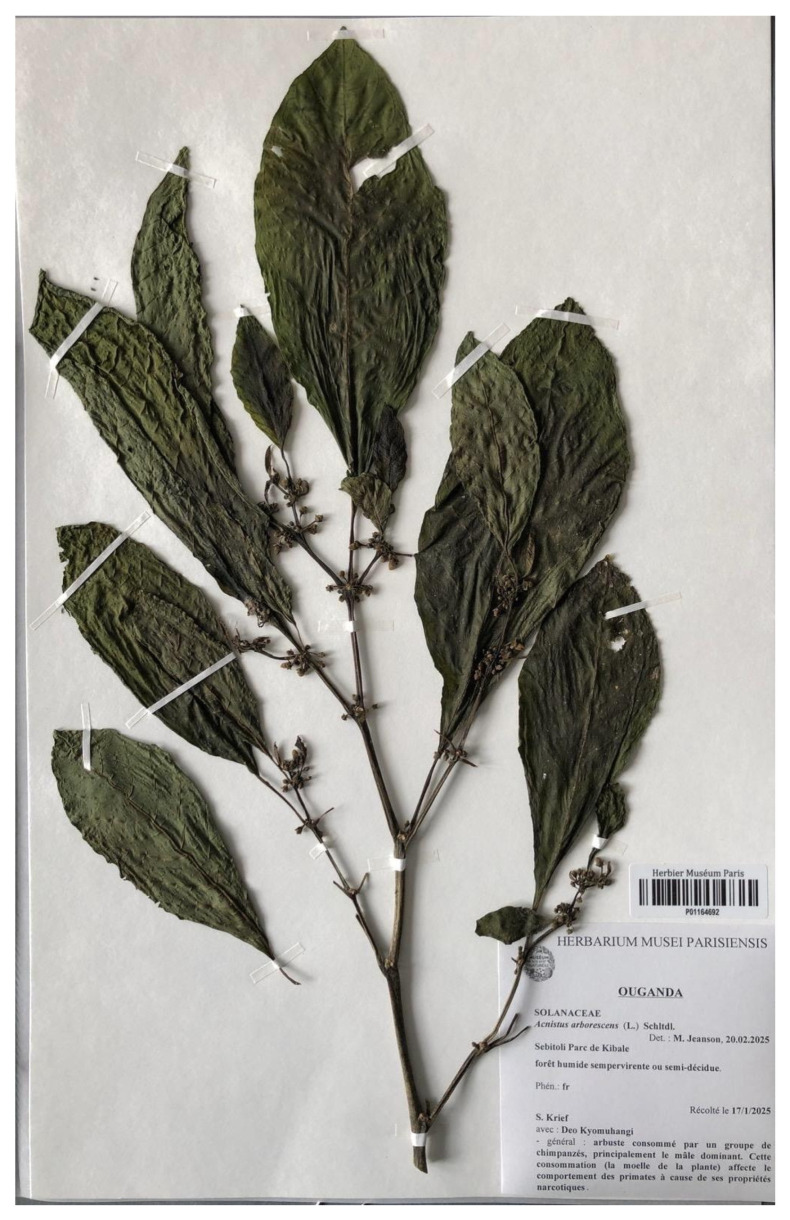
Identified specimen of *Acnistus arborescens* (L.) Schltdl. Linnaea 7: 67. 1832 (syn. *Iochroma arborescens*, J.M.H. Shaw) (credit: Myriam Kourdourli).

Two different methods of consumption of *A. arborescens* were observed based on the age and lignification of the stem of the plant, with almost the same frequency and amount of fresh plant (9.5 g) (1) opening process, where the lignified, aged stem is divided lengthwise and the soft inner part is eaten (14 cases; the mean weight consumed was estimated to be 10.5 g), and (2) peeling process, where the young soft pith is peeled off and consumed (12 cases; the mean weight consumed was estimated to be 9.3 g; see Scheme 1). Both the young pith and the inner part of the lignified stem tasted extremely bitter to human consumers (five SCP members, including one of us, SK). The daily veterinary health check performed on the chimpanzee consumer showed that none of the individuals involved in the 25 observed events showed symptoms of respiratory or digestive illnesses, wounds, or locomotor disorders. The mean body weight of a consumer was estimated at 33 kg. Among unusual foods or unusual activities recorded within an hour before or after the consumption of Angel’s trumpet pith, the adult female GN fed *Piper capense* flowers 6 min after and the adult female KE fed *Cordia millenii* gum exudate 30 min before. An adult male consumed the inner part of a stem 10 min after hunting a black-and-white colobus monkey (*Colobus guereza*) and eating its meat. For four consumers, observers recorded a long resting time (twice 30 min and twice 90 min) after the consumption of *A. arborescens* (Scheme 2).

### 3.3. LC-MS Analyses: Chemical Profile of the Plant Item

**Figure 2:**
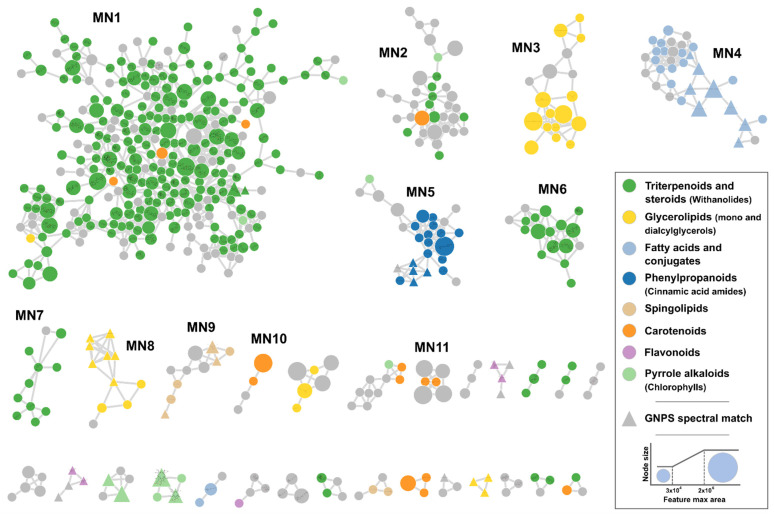
Global molecular networks depicting the chemical space of *A. arborescens* (leaves and pith). The nodes are colored on the basis of the compound class predictions provided by Canopus.

After botanical confirmation of the collected specimen, we used untargeted metabolomics analyses to explore the chemodiversity of *A. arborescens* and provide supplementary clues for its identification. The leaves and pith of the plants were subsequently extracted with ethyl acetate and methanol and analyzed by LC-MSMS. The raw data underwent a peak selection processing step in MZmine 4 to convert these complex results into a manageable and interpretable list of detected metabolites. Spectral data were then organized and visualized as molecular networks using MetGem to group metabolites sharing similar MSMS fragmentation profiles [47,48]. As structurally related compounds share similar fragmentation patterns, similar compounds gather in networks and form clusters of analogs. Metabolites were annotated using GNPS spectral library matching and in silico structure and compound class prediction using Sirius 6 [41]. The resulting networks consisted of 544 nodes (individual ions), themselves grouped into 33 clusters made of at least three nodes (Figure 2). The molecular network was enriched with chemical-structure predictions and compound class to assist in the exploration of the chemical space (see Figure 2).

In addition to some widespread plant chemical families, such as glycerolipids (MN3 and 8), fatty acids (MN4), sphingolipids (MN9), carotenoids (MN10 and 11), flavonoids, and chlorophyll, a large diversity of withanolide derivatives was observed (MN1, 2, 6, and 7). MN1 contains 286 nodes that represent the [M + H]+, [M-H2O + H]+, [M-2H2O + H]+, [M-3H2O + H]+ or [M + NH4]+ withanolide adducts. GNPS MS/MS library matching revealed two hits to Withaferin A, and a significant number of structures (74) were predicted with high confidence by CSI: FingerID (confidence score < 0.5). As they are prone to in-source fragmentation, manual investigation of the data (coelution and mass shift comparisons) allowed for the observation of 136 individual withanolide derivatives (see Supplementary Material Table S2) [38]. Most of them are found mainly in leaves, but few compounds are quite specific to plant piths (see Supplementary Material). A molecular formula has been unambiguously calculated for 112 of them (observation of a coeluting sodium or ammonium adduct together with a protonated ion). These are all C28 withanolides (except one in C27), showing a high degree of isomerism (formula listed in Supplementary Material Table S2). As an example, the molecular formula of Withanolide D or Withaferin A (C_28_H_38_O_6_) returned 14 different observed isomers. Of these 136 compounds, 23 correspond to sulfated withanolides, observed very specifically in methanolic extracts.

The ions found in MN2 and MN6 corresponded to the [M+Na]+ adducts of the protonated ions observed in MN1. Ions shown in MN7 were annotated as triterpenoids/steroids and could represent biological precursors or, conversely, highly degraded derivatives of withanolides. These compounds (MN7) are present in low abundances and are found almost exclusively in plant pith (see Supplementary Material Table S2).

MN5 contained 27 nodes, of which five were predicted as cinnamides (Figure 2). Spectral matching against the GNPS MS/MS library revealed confident hits to feruloyltyramine, feruloylputrescine, and coumaroyl tyramine. Furthermore, CSI: FingerID predicted the structures of 14 cinnamides with high confidence scores.

**Figure 3:**
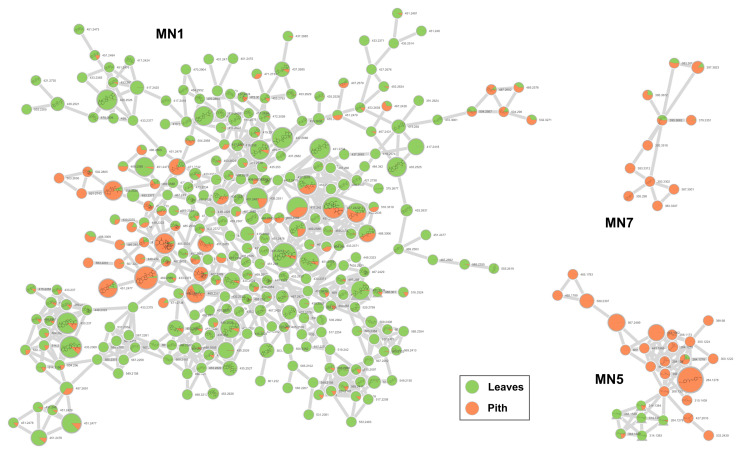
Comparative distribution of withanolides, cinnamides, and triterpenoids in plant tissues. Pie charts represent the relative abundance of each ion, scaled to the peak areas detected in pith (orange) and leaf (green) samples.

Several major compounds appeared to be highly specific to the plant pith, while others were found in greater abundance in the pith compared to the leaves. In the case of withanolides, three compounds were detected exclusively in the pith. Among the 62 compounds observed in both tissues, twelve were more abundant in the pith than in the leaves, while 71 compounds were specifically detected in the leaves (Supplementary Material Table S2 and Figure 3). In general, cinnamides (MN5), as well as triterpenoids and steroids (MN7), appeared to be predominantly associated with plant pith (see Figure 3).

## 4. Discussion

The inner part of the stems of a plant locally called “Angel’s trumpet” and identified as *Acnistus arborescens* is consumed by chimpanzees from the Sebitoli community in the northern part of Kibale National Park, Uganda. A complex process consisting in opening the lignified stems to ingest a small amount of less than 10 g for an average individual body weight of 33 kg has been observed, excluding a consumption “by chance” as a subproduct of the consumption of another item. We could also rule out an error in the selection of the plant species because such a complex process of consumption has not been observed for any other food item in the community. In addition, consumption bouts have been observed in experienced individuals such as the alpha male, five other adult males, five adult females, with some of them consuming it several times. The small quantity consumed and the short average duration of consumption (6 min and 50 s, including opening of the stem) does not appear to be related to resource availability, as in each observed event the consumer left the site while a large quantity of stems remained available on the plant. No feeding competition or food supplanting was observed. Consumption events ended spontaneously and were not interrupted by interference from a conspecific or by any external disturbance. We assume that, on average, less than four events may happen per chimpanzee every year. This rare annual frequency of consumption is consistent with the range of consumption of other natural substances known to have medicinal properties by Sebitoli chimpanzees. It is lower than the consumption of soil and *Trichilia rubescens* leaves, but higher than that of *Albizia grandibracteata* bark and wax and the swallowing of rough leaves observed annually in the same community.

This intriguing behavior related to a plant locally known as toxic and repellent to insects led us to collect and survey the plant and confirm its botanical identification as *Acnistus arborescens* (L.) Schlecht (Solanaceae), a medicinal plant common to South and Central America [52]. Surprisingly, *Acnistus arborescens* has never been documented in Africa, and no specimens outside its native range are recorded in the main reference collections of the national herbarium in Kampala or at the National Museum of Nature d’Histoire naturelle (Paris, France), which contains more than 8 million specimens.

*A. arborescens* is popularly known in Brazil as “fruto-de-sabiá”, “esporo-de-galo-falso”, and “marianeira”, and is found throughout the Brazilian territory, from the northeast to the south, within the entire phytogeographical domain of the Atlantic Forest [53]. In local Brazilian medicine, it is also known as “Tabak Djab” (meaning “Devil’s tobacco”). Several uses of the plant have been reported, including sedative effects on the central nervous system as a narcotic [54,55,56]. Extracts of *A. arborescens* exhibited pharmacological activities such as antiparasitic and cytotoxic properties, and have been investigated for the treatment of atherosclerosis and degenerative diseases [56,57,58]. Previous investigations of *A. arborescens* specimens from America have mainly led to the isolation of withanolide-type compounds, underlining their prominence in the chemical profile of the species [59,60,61,62,63]. Withanolides exhibit a wide range of biological activities, including neuroprotective, antitumor, anti-inflammatory, immunomodulatory, and cardioprotective activities [64,65]. The role of withanolides in reducing oxidative stress within neurons has been demonstrated in multiple animal models of different disease processes, as well as the neuroregenerative role of several withanolides in functional locomotor improvements [66]. Treatment with withanolide extract demonstrated a significant anxiolytic effect at a low dose, similar to that of benzodiazepines. In vivo studies on rodents demonstrate a consistent amelioration of stress and anxiety, and in humans, the quality of sleep was improved [67,68,69,70]. Such effects may be consistent with the narcotic properties reported by the Brazilian literature of traditional medicine [66,67,68]. Beyond their bioactive potential, the identified withanolides serve as a chemotaxonomic marker underscoring the affiliation of *A. arborescens* within the Solanaceae, a family rarely consumed by chimpanzees. These anxiolytic and narcotic effects, even if they are experienced by chimpanzees, are difficult to quantify in chimpanzees living in their natural environment. Unlike a pathological condition with clearly measurable symptoms, such as cough or diarrhea, that could explain the selection of a plant part, the biological activities associated with the consumption of *A. arborescens* plant part are difficult to demonstrate objectively.

The metabolomics analyses we conducted fully support the identification established through botanical analysis. They also confirmed that leaves and pith have a different chemical profile, with three withanolides specific to the pith part of the species and 12 withanolides more abundant in pith than in leaves, and with cinnamides, triterpenoids, and steroids predominantly present in the pith.

Observation or isolation of sulfated withanolides, as the 23 observed in the methanolic extracts prepared during this study, is scarce, but has been described in *A. arborescens* [63]. O-sulfated withanolides isolated from *A. arborescens* had anti-inflammatory activities in vitro and showed cytotoxic properties [63].

Five compounds were predicted to be cinnamides. Cinnamic acid amides are considered a defense mechanism against various environmental stresses, already reported in *Acnistus species* [71,72,73,74]). They are known to exhibit a broad spectrum of biological activities, encompassing anticancer, antitubercular, anti-diabetic, antimicrobial, antiviral, anti-inflammatory, and neuroprotective properties [75].

The presence of compounds specific to or more abundant in plant pith may provide insight into the intriguing behavior of chimpanzees. They peel or open woody stems to consume the inner tissue, even though leaf consumption would be easier, faster, and more nutritive.

Given the rarity of this plant material in the habitat, the small amount consumed relative to the effort required, and the fact that typically only a single often-experienced individual engages in this behavior, this pattern is unusual and suggests that such selectivity may be related to a distinctive taste and/or the specific chemical composition of the pith. However, the chimpanzee only consumes 10 g, while a large amount is available on the plant, suggesting potential toxicity related to the greater amount consumed.

Since no chimpanzee that consumed the stems of *A. arborescens* exhibited any specific symptoms during the daily health check, it may be used to (i) cure non-visible symptoms, such as fever, pain, or inflammation not related to external injury; (ii) as prophylaxis; or (iii) because the plant and the withanolide compounds are known for their psychoactive properties, they could provide some anxiolytic effects to the consumer. Consumption of two plant parts also having bioactivities was observed shortly before or after ingestion of *A. arborescens* and may indicate a search for medicinal properties by the chimpanzee. *Cordia millenii* Bak. (Boraginaceae) is used in Southwest Nigeria to cure ache and pain disorders, and the essential oil obtained from the root bark showed anti-inflammatory and high antinociceptive activities [76,77], while a review by Kamsu and Ndebia [78] shows that extracts and compounds isolated from *Piper capense* L.f. (Piperaceae) have interesting anticancer, antibacterial, antimalarial, hypoglycemic, antiepileptic, and antidepressant activities. In another case, a consumer participated in a stressful activity (hunt) 10 min earlier. In four cases, consumers had a very long rest from 30 min to 90 min after consumption. We cannot exclude the possibility that the consumption of these stems may induce relaxing or anti-stress effects in chimpanzees, as similar effects have been reported in in vivo studies, including reductions in anxiety in rodents and improvements in sleep quality in humans [68,69,70]. However, additional observations are necessary to support the hypothesis that this behavior reflects medicinal use or at least biological effects.

Such a report emphasizes the great adaptability of nonhuman animals toward novelty and their ability to discover and use unusual properties, which is not restricted to nonhuman primates, such as, for example, the use of cigarette butts, which reduced nest anti-ectoparasites by urban birds [79]. In nonhuman primates, innovative and flexible feeding behaviors can occur in response to habitat disturbance, as highlighted in the review by Nowak and Lee [80]. For example, the Udzungwa red colobus (*Procolobus gordonorum*) in Tanzania inhabits and feeds in rubber (*Hevea brasiliensis*) plantations, whereas the endangered lion-tailed macaque (*Macaca silenus*) in the Anamalai Hills, India, subsists on exotic food items such as tea, eucalyptus, and coffee [81]. When confronted with new foods, the responses of chimpanzees usually combine a mixture of curiosity and caution [82]. Although caution and bitter-taste aversion were selected to prevent any risk of poisoning with toxic compounds from plants, social learning opportunities can favor the adoption of a novel low-palatable vegetative plant part [83,84].

The way and why the *A. arborescens* species was introduced to Uganda, and specifically in the protected area of Kibale National Park, remain unknown. On the basis of the plant’s uses in America, it can be hypothesized that *A. arborescens* was introduced to Africa either for its ornamental properties or even for its narcotic effects. In the case of ornamental or recreational use or traditional medicine, one would expect the plant to be found near human dwellings and not in the forest. However, dissemination by birds, and then by primates and elephants, is possible, if they consume the fruits. Chimpanzees and elephants have never been observed to consume fruits to date. It should be noted that the same question of how the plant was introduced in India was questioned and is still unresolved [85]. The authors mentioned their concern about the invasive nature of the species because of its ability to inhibit the growth of nearby plants and colonize in a newly introduced area.

## 5. Conclusions

Long-term monitoring of chimpanzees that integrates behavioral, health, and contextual data may further elucidate the motivations and potential effects of *Acnistus arborescens* ingestion. Such monitoring may also assess whether resting time changes significantly following ingestion. Additionally, it could determine whether energetically or socially stressful activities—such as territorial patrols, intercommunity encounters, conflicts, incursions into cultivated areas, or hunting—precede consumption and whether these activities could explain a specific need for relaxation.

Such findings may encourage the reporting of similar consumption events at other sites and in chimpanzee communities, thus contributing to a greater understanding of this unusual behavioral phenomenon. This study highlights the practical importance of continuing these observations, both to confirm the distribution of this plant in Uganda and across Africa, and to investigate how it spreads out of its native geographical range and its potential invasive nature, as well as its uses and effects on wildlife and humans, given its toxicity and/or anxiolytic properties or any medicinal properties.

## Figures and Tables

**Table 1:** Observed consumptions by chimpanzees and estimated individual annual consumption frequency for items of low or no nutritional value (based on 2024 observations).

Estimated Individual Annual Consumption Frequency (Number/Year)	95% Confidence Interval	Per-Event Probability of Consumption	Observed Consumptions (Total Number)	Mean Event Duration	Item
3.45	[3.16 × 10^−5^, 8.01 × 10^−5^]	5.62 × 10^−5^	20	6 min 9 s	*Acnistus arborescens*
3.84	[1.34 × 10^−4^, 2.75 × 10^−4^]	2.05 × 10^−4^	32	14 min	*Trichilia rubescens*
0.72	[7.67 × 10^−7^, 6.90 × 10^−6^]	3.84 × 10^−6^	6	1 min 24 s	*Albizia grandibracteata*
0.84	[9.80 × 10^−7^, 6.58 × 10^−6^]	3.78 × 10^−6^	7	1 min 11 s	rough leaves

## Data Availability

Data are available to interested researchers upon request.

## Supplementary Materials

The following supporting information can be downloaded at: https://www.mdpi.com/article/10.3390/ani16132031/s1, Table S1: Krief_Acnistus_behavior-chimps; Table S2: Acnistus_Leaves&pith; Supplementary Material S1: phytochemistry_methods.
