# Supplementary material for: Chimpanzee-Guided Discovery of a Non-Native Bioactive Plant
Source: Animals (Basel). 2026 Jul 2;16(13):2031. doi: 10.3390/ani16132031 (PMC13359941; doi:10.3390/ani16132031)
Supplement: Supplementary file 1 [file animals-16-02031-s001.zip › animals-4320261-SI-phytochemistry_methods.pdf]

MDPI animals

Supplementary information

1. MZmine processing parameters

mzML files were processed using MZmine 4.4.3 [36]. Mass detection was carried out using a noise level of 400 counts for MS and 100 counts for MSMS dimensions. The LC-MS chromatogram builder was used with a minimum consecutive scan of 4, a minimum intensity for consecutive scans of 500, a minimum absolute height of 1500, and a tolerance of m/z of 10 ppm [37]. The local minimum feature resolver was used with the following standard settings: chromatographic threshold, 80%; minimum search range RT (absolute), 0.06 min; minimum absolute height, 1500; minimum ratio of peak top/edge, 1.8; maximum duration range (min), 0.0–1.0; minimum scans, 4. MSMS scans were paired using an MS1 to MS2 precursor tolerance of 20 ppm and the feature edges were used as the RT filter. Isotopes were grouped using the 13C isotope filter with an m/z tolerance of m/z of 5 ppm, an RT tolerance of 0.03 min, a monotonically decreasing isotope pattern, a maximum charge of 2, and the most intense peak as the representative isotope. Further isotope signals were searched using the isotopic peaks finder module with an m/z tolerance of 5 ppm. Peak alignment was performed using the joint aligner module (m/z tolerance, 5 ppm; weight for m/z, 1; RT tolerance, 0.05 min; weight for RT, 1). The feature list was filtered to retain only features that include MSMS scan and was filled with the peak finder module (m/z tolerance, 4 ppm; RT tolerance, 0.1 min; minimum scans, 4). The duplicate peak filter (m/z tolerance, 2 ppm; RT tolerance, 0.02 min) was applied. The ion adducts were then grouped and annotated using metaCorrelate feature grouping and ion identity networking modules [38].

2. Sirius compound annotation details.

Molecular formulae, chemical structures, and compound classes were annotated using Sirius 6.0.7 [41]. Molecular formulae were calculated based on isotopic patterns and fragmentation tree analyses with an MS2 mass accuracy parameter set to 4 ppm [42,43]. Formula predictions were further improved using the ZODIAC module [44]. CSI: FingerID was used for in silico structure annotation [45]. Only biological databases were considered for the prediction of the structure. The Canopus module was also used for compound class annotation [46].
